# Classifying Multigraph Models of Secondary RNA Structure Using Graph-Theoretic Descriptors

**DOI:** 10.5402/2012/157135

**Published:** 2012-11-11

**Authors:** Debra Knisley, Jeff Knisley, Chelsea Ross, Alissa Rockney

**Affiliations:** ^1^Institute for Quantitative Biology, East Tennessee State University, Johnson City, TN 37614-0663, USA; ^2^Department of Mathematics and Statistics, East Tennessee State University, Johnson City, TN 37614-0663, USA

## Abstract

The prediction of secondary RNA folds from primary sequences continues to be an important area of research given the significance of RNA molecules in biological processes such as gene regulation. To facilitate this effort, graph models of secondary structure have been developed to quantify and thereby characterize the topological properties of the secondary folds. In this work we utilize a multigraph representation of a secondary RNA structure to examine the ability of the existing graph-theoretic descriptors to classify all possible topologies as either RNA-like or not RNA-like. We use more than one hundred descriptors and several different machine learning approaches, including nearest neighbor algorithms, one-class classifiers, and several clustering techniques. We predict that many more topologies will be identified as those representing RNA secondary structures than currently predicted in the RAG (RNA-As-Graphs) database. The results also suggest which descriptors and which algorithms are more informative in classifying and exploring secondary RNA structures.

## 1. Introduction

The need for a more complete understanding of the structural characteristics of RNA is evidenced by the increasing awareness of the significance of RNA molecules in biological processes such as their role in gene regulatory networks which guide the overall expressions of genes. Consequently, the number of studies investigating the structure and function of RNA molecules continues to rise and the characterization of the structural properties of RNA remains a tremendous challenge in computational biology. RNA molecules are seemingly more sensitive to their environment and have greater degrees of backbone torsional freedom than proteins, resulting in even greater structural diversity [1]. Although the tertiary structure is of significant importance, it is much more difficult to predict than the tertiary structure of proteins. Advances in molecular modeling have resulted in accurate predictions of small RNAs. However, the structure prediction for large RNAs with complex topologies is beyond the reach of the current *ab initio* methods [2].

A coarse-grained model to refine tertiary RNA structure prediction was developed by Ding et al. [2] to produce useful candidate structures by integrating biochemical footprinting data with molecular dynamics. Although the focus is on tertiary folds, their method uses information about RNA base pairings from known secondary structures as a starting point. This, coupled with the understanding that the RNA folding mechanisms producing tertiary structure are believed to be hierarchical in nature, implies that much can be achieved by discovering all possible secondary structural RNA topologies.

Given the primary sequence of an RNA molecule, there are a number of algorithms and tools available to predict the most likely set of resulting secondary structures. The most widely used algorithms such as Zucker's Mfold [3] and Vienna RNAfold [4] typically base their predictions on the minimum free energy paradigm. While these algorithms have been highly beneficial, it is not always the case that the predicted structure with minimum free energy is the correct one and consequently some suggest that the actual RNA secondary structure may not have a minimum global free energy, only local ones [5]. Other means of characterizing the topology of secondary RNA structures are still an active avenue of pursuit.

The graph representations used in this work can be found in the database RAG: RNA-As-Graphs [6]. Secondary RNA structure is modeled by two graph-theoretic representations in the database resource RAG (see [6] for additional details on the differences between the two). In one of these representations, regions of the secondary structure that consist of unpaired bases such as junctions, hairpins, and bulges are represented by vertices. The connecting stems are represented quite naturally as connecting edges. The resulting graph is a connected, acyclic graph, that is, a tree. One advantage of this representation is the fact that trees have been highly studied in the graph theory thereby providing a wealth of information about the model. For instance it is known by the generating function developed by Harary and Prins [7] exactly how many distinct trees can be constructed for a given number of vertices. This allows the entire space, that is, all possible configurations, to be considered. Unfortunately, secondary RNA structures containing a pseudoknot cannot be represented as described above by the tree model. If, however, the model is reversed and stem regions are represented as vertices and connecting strings of unpaired bases as the edges, all secondary RNA structures can be now be modeled, including those that contain a pseudoknot. This representation is called the dual graph in the RAG database. The resulting dual graph however is no longer a simple graph; instead this method produces a multigraph. Unlike a simple graph, a multigraph can have more than one edge connecting a pair of vertices. And, unlike simple graphs, multigraphs have not been as highly studied in the theoretical setting. In previous work [8], the authors of this paper, together with Koessler et al., capitalize from the knowledge afforded by the graph theory and exploit the tree representation of the secondary RNA structure to build a predictive model that identifies whether a given tree structure is RNA-like or not RNA-like. In this work, we now consider the dual graph representation.

In particular, all possible dual graph representations of orders 2, 3, and 4 are given in the RAG database and the corresponding structures are classified as either (a) representing a known structure or (b) not representing a known structure. Those not representing a known structure are further classified as either likely to represent a structure in the future, that is, having the characteristics of RNA structure making it likely that such a structure will be identified at some point, or not RNA-like in structure. For the dual graphs of order 5, the database contains 18 structures that have been identified and states that there are 108 possible dual graphs of order 5. This number was determined by a graph growing algorithm. Eighteen of these 108 graphs are verified as representing existing RNA structures and the remaining 90 structures are classified as either RNA-like or not RNA-like in the most recent update for the database by Izzo et al. [9]. This update describes two methods by which the unverified structures are classified. The Laplacian eigenvalues for each structure were transformed using a linear regression to obtain two values for each structure and then these values were applied in two clustering algorithms, namely, a partitioning method called PAM and a *k*-nearest neighbor algorithm [9]. They state that 63 are RNA-like and 36 are not and that 45 are RNA-like and 45 are not RNA-like by the two methods, respectively. Since only 18 structures are provided in the database, our objectives were to (1) combinatorially analyze the structures of the 90 dual graphs of order 5 not in the RAGs database and (2) predict which of those 90 dual graphs of order 5 are RNA-like in structure via graph-theoretic information from chemical graph theory and mathematical graph theory. 

Our findings differ significantly from those of Izzo et al. [9]. We find by using a combinatorial algorithm to construct all possible graphs with the given constraints that there are 118 instead of 108 possible dual graphs of order 5. Furthermore, we show that indeed almost all of the structures in the database with 5 vertices are RNA-like instead of approximately half as indicated in [9]. We feel that this is not too surprising. In the earlier version (2004) of the database, for instance, 8 of the 30 possible tree graphs were classified as not RNA-like, but in the updated version (2011), only 3 graphs are listed as not RNA-like. We expect that the remaining 3 topologies will be verified as RNA topologies as more RNA molecules are found. For example, genome-wide mapping of conserved RNA secondary structures reveals evidence for thousands of functional noncoding RNAs [10]. In the following sections, we discuss the dual graph representation and the graph-theoretic measures that we use. We then discuss the analysis and training together with the results.

### 1.1. The Dual Graph Representation of Secondary RNA Structure

Gan et al. [6] have used both tree graphs and the corresponding dual graphs which results in a multigraph representation of RNA secondary structures. Here, however, we will restrict our study to the multigraph representations of RNA secondary structures. As mentioned previously, the dual graphs can represent all types of RNA secondary structures, including the complex pseudoknot structures. When representing an RNA structure with a dual graphs, a vertex is used to represent stems (two or more complementary base pairs), and circular edges are used to represent the RNA motifs (hairpin loops, bulges, internal loops, and junctions). Dual graphs may contain multiple edges and loops; however, neither of these structures is required. Since a double-stranded RNA stem is connected to at most 2 strands on each side, every vertex *v* must have at most degree four. In fact, all vertices are of degree 4 except either (a) one of degree 2 or (b) two of degree 3. It follows that dual graphs of order *n* are of size 2*n* − 1 [6]. Given these constraints, we use a constructive graph algorithm to enumerate the number of dual graphs of order five. These 118 graphs may be found in Figure 6.

### 1.2. Previous Results for the Dual Graph Model

The dual graph representation with 4 or fewer vertices was used in a previous work to train an artificial neural network (ANN) to recognize a dual graph as having the structural properties of secondary RNA [11]. In particular, we quantified the structures using graph invariants from graph theory and molecular descriptors from chemical graph theory and then used a multilayer perceptron artificial neural network to verify the findings in the RAG database regarding the classification of the dual graphs of order four. A set of ten structures that have been verified as RNA-like were chosen randomly from the set of 11 RNA-like graphs of order four. These ten graphs, in addition to the ten classified as not RNA-like, comprised the training set for the ANN. All graphs that were classified to be RNA-like in the database were predicted to be RNA-like by the neural network. However, one of the graphs whose structure represents a known topology was predicted with much lower probability than the other graphs in the set. Since this earlier work, the RAG database has been updated and a dual graph considered to be not RNA-like has since been changed to RNA verified [9]. This particular structure is similar to the structure that the neural network predicted to be RNA-like, but with lower probability. Given the updated information in the RAG database, we can now remove the incorrectly predicted structure from the training set and expect our results to confirm the new information. Thus, even with incorrect information in the training set, the graph-based measures were sufficient to characterize the topology of the RNA-like dual graphs of order 4.

We extend these findings to the dual graphs of order five. For this work we do not use the predicted classifications of the RAG database. We use only the verified structures in the database of which there are 18 of order 5 as well as 17 of order 4. We refer to these verified structures as RNA graphs. We consider the remaining 13 graphs of order 4 and 100 graphs of order 5 as unclassified structures.

## 2. Graph-Theoretic Measures for the Dual Graphs

As stated previously, the dual graph representation method of the RAG database results in a multigraph. We began by writing a program in the computer language Python which generates the 30 multigraphs of order 4 and the 118 multigraphs of order 5. This program realized edgeless graphs as networkx [12] multigraph structures and then generated edges in accordance with the secondary RNA structural constraints. Several algorithms to calculate topological indices and graph invariants were also written in Python based on the networkx graph object.

In order to draw upon the wealth of graph-theoretic measures to quantify the topologies of the RNA model, we note that the majority of such measures is defined for simple graphs, and simple graphs do not have multiple edges nor do they have loops. Given that the dual graph representation has both, we therefore determined the line graph of each of the dual graphs and we use the line graph representation to determine the graphical measures of the topologies such as the clique number (both edge and vertex), independence number, anddiameter and domination numbers. The line graph of a graph *G* is defined as the graph whose vertex set is the edge set of *G* and two vertices are adjacent in the line graph if the corresponding edges in *G* are incident. Thus the vertices in the line graph correspond to the regions in the RNA molecule with unpaired bases. Using the line graph of the dual graph allows quantification of the structural properties of the RNA molecule with graph-theoretic descriptors, even those containing pseudoknots. An algorithm for generating the line graph of a multigraph was also written in Python, and this algorithm was used to generate the 30 + 118 line graphs of the multigraphs of orders 4 and 5. The multigraphs and line graphs were verified by the authors via a comparison to the RAG database and by manual inspection and reconstruction.

To calculate the graph-based measures, we used the GraphTheory package in Maple, the networkx package in Python, and the network analysis plugin in Cytoscape 2.8.2 [13]. Many invariants—such as diameter, radius, and clique numbers—were calculated either in all 3 or in 2 of the 3. This allowed us to verify the results of each software tool or to identify any variations in the graph invariant and/or topological index techniques. Most but not all of the measures we used can be found in at least one of the three tools mentioned above. In order to calculate a number of the measures, especially the topological indices, we need to determine the distance matrix of the graph. In a simple graph, the distance from a vertex *u* to itself is zero. However, with the presence of a loop, we considered three possibilities. One is the standard distance matrix with zeros down the diagonal. In the second case, we place either a zero or a one, depending on whether the vertex has a loop. In the third case, we not only modify the diagonal but also if the shortest path traversal includes a vertex with a loop, we include the loop in the edge count of edges encountered. Thus we are requiring any traversal to include a loop when encountered. We also modified the Balaban index, motivated by recent results using random walks on graphs. To find the distance between two vertices *u* and *v* in a dual graph, observe that if *u* is a vertex with a loop and if there are two edges between *u* and *v*, then the following options arise:

- one of the edges from *u* to *v* is traversed;
- the other edge from *u* to *v* is traversed;
- the loop is traversed followed by a traversal of one of the edges.

There are four possibilities, so each traversal is assigned an equal weight of 1/4. The shortest route is the traversal of one edge which can happen in two ways. Thus the distance from *u* to *v* is 1/2.

We subsequently calculated approximately 100 invariants and indices of the multigraphs and line graphs using the 3 graph theoretic software tools mentioned above, some with slight modifications to account for the presence of loops and multiple edges. The invariants were normalized with respect to the values of the graphs that are verified as representing a known RNA secondary structure.

## 3. Assessing the Graph-Theoretic Measures as Descriptors of RNA Topology

 The total invariants were divided into 3 categories—topological indices, graph-theoretic invariants, and measures on line graphs. In order to compare the efficacy of an invariant or index in discriminating between the RNA graphs and the remaining graphs, the invariants were normalized with respect to the RNA graphs of orders 4 and 5, respectively. In particular, for each invariant or index, we calculated the mean and standard deviation of the RNA graphs of order 4, after which we used this mean and standard deviation to normalize all the values for graphs of order 4 of the given invariant or index according to the formula

(1) Inormalized=Iobserved−MeanRNA graphsStDevRNA graphs.

**Figure 1:**
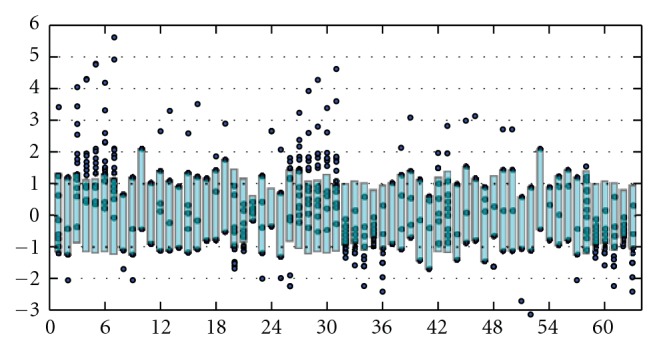
Topological invariants for RNA multigraphs of order 4.

Figure 1 shows the 10% percentile to 90% percentile of each normalized index/invariant in the topological indices collection as a rectangle. The mean is zero and the standard deviation is one for the given index across the RNA graphs of order 4. The values of the unverified graphs of order 4 are shown as points, so that a point inside the given rectangle is between the 10% and 90% percentiles for that index. The dotted lines correspond to the numbers of standard deviations from the mean. In general, if the values of the unverified graphs are close to the values of the verified graphs (i.e., if the dots are all on or inside the rectangle for a given invariant), then this invariant will not be useful as a factor in a machine learning classifier. For example, invariants 12–18 are poor predictors of RNA-like versus not RNA-like simply because there is not enough variation among the values for all the multigraphs of order 4. A support vector machine, a neural network, and logistic regression trained on the multigraphs of order 4 using invariants 12–18 were no better classifiers than was the uniformly random assignment to different classes, as evidence by the Receiver Operating Characteristic analysis in which the area under the curve for each method was approximately 0.5.

**Figure 2:**
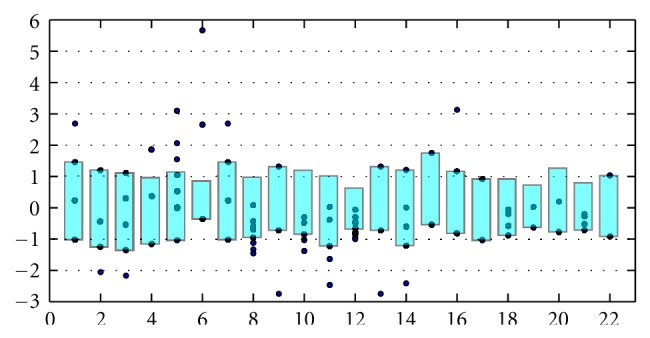
Variations on the Balaban index.

In contrast, invariants 2 through 7 in Figure 2 are variations on the Balaban index for the graphs considered as simple graphs, and invariants 24–32 are variations on the Balaban index for the graphs considered as multigraphs. Like invariants 11–19, there is insufficient discrimination in each of the remaining topological indices, which includes eigenvalues of the Laplacian, the clustering coefficient, variations on the Weiner index, variations on the Randic index, variations on the Platt index, various measures of centrality, associativity, and connectivity, topological coefficients, and stress. Unfortunately, even though the Balaban indices and their variations have better discriminatory ability, they alone do not characterize between those graphs verified as RNA and those that are unclassified.

**Figure 3:**
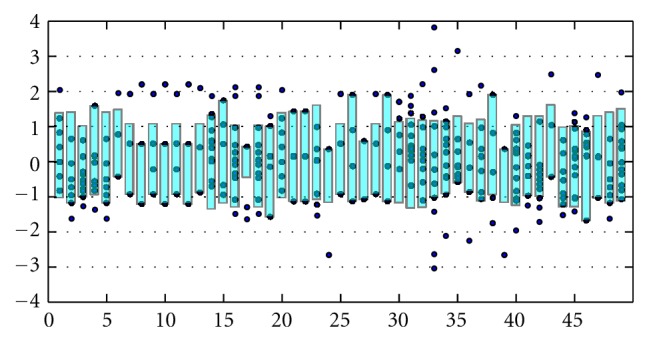
Line graph invariants.

First, we find that variations on the clique number yield another factor with the ability to discriminate between the RNA graphs and the unclassified graphs. Observe invariants 4, 5, and 6 in Figure 2. Second, invariants and indices based on the line graphs retain more of the information contained in a multigraph than does a simple graph interpretation of a multigraph, while additionally allowing standard algorithms to calculate the invariants. For example, in Figure 3, invariants 7 through 12 are the chromatic index, the chromatic number, the circular chromatic index, the circular chromatic number, and the edge chromatic number, respectively, of line graphs of order 4. 

Invariants 16 through 18 are variations on the clustering coefficient, and invariant 33 is the network centrality of the line graphs. Invariant 21 is the diameter, invariant 27 is the independence number, and invariant 28 is the maximum degree of the line graphs. It is interesting to note that the Balaban index of the line graphs, invariant 4, is not a good discriminator.

## 4. Results

There are 18 multigraphs of order 5 that have been verified so far. The consensus across several techniques—including clustering, machine learning, and nearest neighbor analysis—and across several different combinations of invariants and indices indicate that most, if not all, of the unverified graphs are RNA-like.

For example, a simple machine learning scheme is that of choosing one unverified graph to be in class 0 while the 18 verified are in class 1. The neural network is then trained and the remaining unclassified RNA graphs are tested. Overwhelmingly, most if not all were classified as being in the same class as the 18 verified—that is, assuming only one non-RNA-like graph confirmed that all the graphs are RNA-like independent of which unverified graph was chosen to be RNA-like.

Regression, neural network, and support vector machine analysis similarly confirm the observation above. Nearly all the graphs of order 5 are predicted to be RNA-like in each run, and the ones that are predicted to be not RNA-like change from one run to the next.

Subsequently, we applied several different classifier/clustering techniques to graphs of order 5. Many different subsets of invariants and indices were used, but the invariant set suggested by the analysis above—as well as the one that produced the best results—was the following:

- Four to eight variations of the Balaban index for multigraphs;
- Clique numbers;
- Chromatic numbers of the line graphs;
- Edge chromatic number of a line graphs;
- Clique numbers of the line graphs;
- Diameters of the line graphs;
- Independence numbers of the line graphs;
- Maximum degrees of the line graphs.

Likewise, many different partitions of the total data were used, including the restriction to order 5 graphs known to be RNA-like. Results were consistent across these variations.

**Figure 4:**
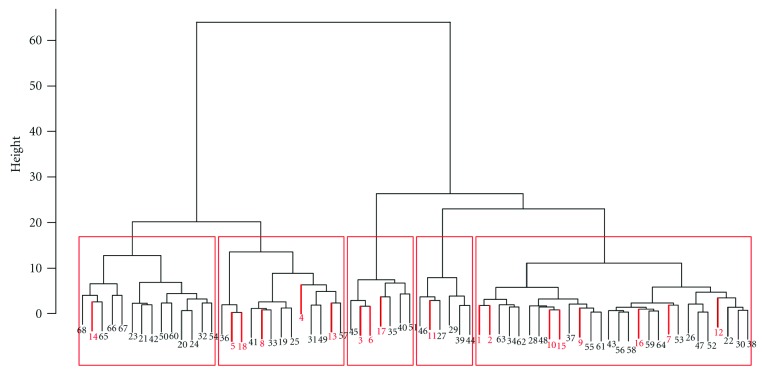
Clustering of the 50 graphs most distant from the 18 verified as RNA-like (in red).

In particular, clustering tended to group all unverified graphs of order 5 with the 18 verified to be RNA-like (see Figure 4). To further investigate, we ranked the 100 unverified graphs using nearest neighbor analysis, and then we clustered in two groups—the 50 closest to and the 50 furthest from the 18 verified structures. The 50 closest to the 18 verified formed a single cluster with the 18 (using biclustering and hierarchical clustering in the statistical language *R*). The 50 furthest from those verified likewise clustered with the 18, but in a somewhat interesting manner. Having determined a 5-cluster scheme to be the best, we found that one cluster contained only one of the 18 verified graphs of order 5, and this graph (105 in our numbering) was both a large distance from the other 17 and had no more than an *r* = 0.49645 correlation with any of the other verified graphs. 

Moreover, this was a rather large cluster containing 14 graphs of order 5, and, likely, if there were any graphs of order 5 that are eventually deemed to not be RNA-like, they would come from this cluster. However, the results seem to further support an interpretation of all the graphs of order 5 being RNA-like.

### 4.1. Data Domain Description

This interpretation motivated us to consider the problem to be a *data domain description* problem, also known as a one-class classification problem. In particular, rather than predict whether or not a graph is RNA-like, we instead explore the degree to which the 18 verified graphs typify the entire class of RNA-like graphs.

To do so, we use a “cognitive learning” approach in association with an artificial neural network [14]. While this is typically performed with a support vector machine [15, 16], our goal is to examine how the unverified RNA multigraphs of order 5 are distributed about the 18 verified multigraphs. In particular, the graded response of the neural network can be used to implement a genetic algorithm for successively refining the learning set of a neural network.

Suppose that we are given a training set *P* that contains examples from only one class of data along with a test set *S* of unclassified data that may or may not contain examples from another class. The method begins with a prior assumption: patterns that are many standard deviations away from any pattern in the training set form at least one other class of patterns. This assumption is used to generate an initial “negative example set”, *N*, of *large σ patterns*, after which the algorithm proceeds as follows.

1. Train the neural network with *P* ∪ *N*.
2. Classify the set *S* with the neural network. The classifications are numbers in [0,1].
3. Use the Receiver Operating Characteristic (ROC) or similar method to find the optimal threshold for distinguishing between patterns in *N* and patterns in *P*.
4. Choose some number *n* of the highest scored patterns in *S* to be moved into *P*, being careful to stay above the threshold in step 3.
5. Choose some number *m* of the lowest scored patterns in *S* to be moved into *N*, being careful to stay below the threshold in step 3.
6. Move the *q* patterns in *N* and the *r* patterns in *P* not correctly classified into the set *S*.
7. Eliminate the large *sigma* patterns (after the first iteration).

The algorithm proceeds either until *S* is empty, or through some set number of iterations. In practice, changes to *N*, *P*, and *S* are based on upper and lower thresholds based on the results of step 3.

Although the process is closely supervised in practice, the goal is to mimic the cognitive learning process of regrouping via reinforcement. Ideally, if there is more than one class in the initial *P* ∪ *S* set of patterns, then a two-class classifier will emerge in the process. If there is only one class in *P* ∪ *S*, then the algorithm will proceed until all (or in practice, most) of the patterns initially in *S* are in *P* and all the patterns in *N* are large *sigma* patterns. Moreover, the rate at which a pattern moves into *P* can be used as a measure of how close those patterns are to those in *P* itself.

The algorithm was tested on several standardized data sets from various sources and repositories. When there are two or more distinct classes, which is to say that *S* contains one or more classes distinct from *P* initially, then the algorithm stabilizes to a distinct non-*P* class containing *N* in each iteration. When there is only one class overall, then the set *N* is eventually empty. Within a domain description problem, the final set of patterns in *N*,by which we denote *N*_*f*_, is significant in that it differs the most in some sense from the initial *P* class.

The latter was the case with the classification of the RNA-like multigraphs of order 5. In each of 10 trials, the set *N* became empty after a relatively few number of iterations. However, the final set *N*_*f*_ differed only slightly between trials and is accurately represented by 9 graphs. The graphs in *N*_*f*_ likewise were quite similar, in that each of the graphs contained a triangle with at least one vertex of degree 4.

**Figure 5:**
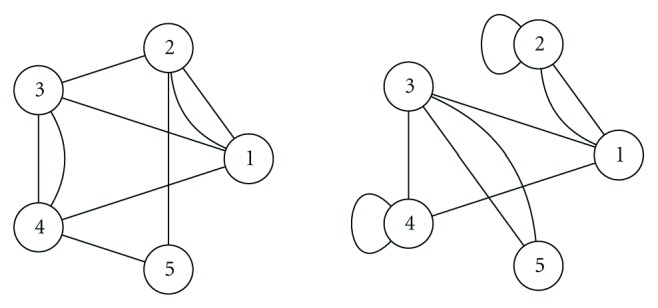
Two graphs from *N*_*f*_.

**Figure 6:**
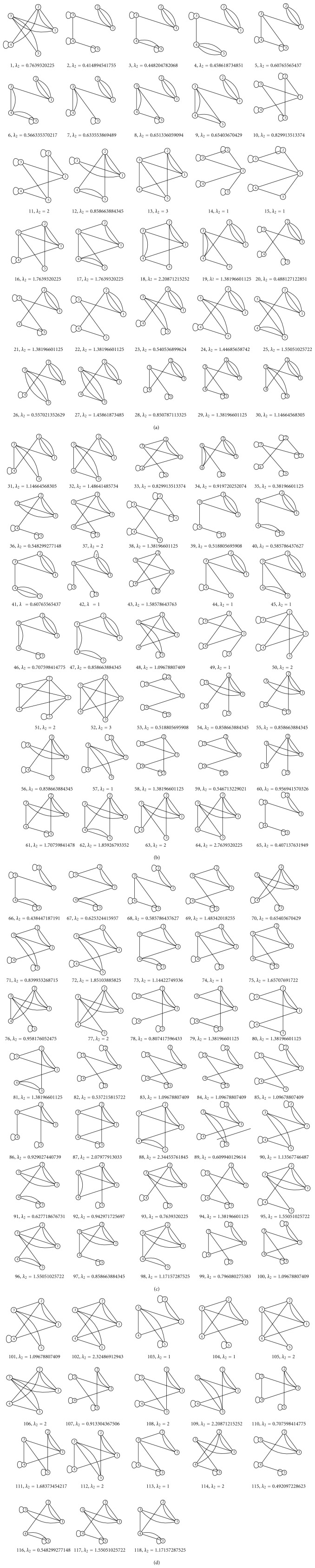
The 118 multigraphs of order 5.

Moreover, as *N* began to lose graphs in the algorithm above, the graphs that tended to remain the longest were those graphs containing triangles with at least one vertex having degree 3 or 4, as illustrated in Figure 5. Finally, the set *N*_*f*_ had no discernible relationship to the clustering or nearest neighbor results discussed earlier, further suggesting that all the multigraphs of order 5 are RNA-like.

## 5. Conclusion

The most reasonable conclusion of this extensive analysis is that all the graphs of order 5 are likely to be verified as RNA structures. Indeed, across several variations of nearest neighbor analysis, machine learning, and clustering techniques using a variety of subsets of different graph invariants and topological indices, we consistently found that more than 90% of the unclassified graphs were closer to one of the 18 already verified as an RNA structure than the 18 were to each other.

This result is not surprising. Initial classification of the graph structures in the database RAG classified more than half of the dual graphs of order 4 as not RNA-like in structure. However, as more secondary RNA structures were identified, an update to the RAG database now predicts only a third to be not RNA-like in structure [9]. We predict that as the number of new motifs continues to increase, eventually almost all structures will be classified as RNA-like or verified as an RNA topology. Does this mean that the graph model in the database is too coarse to be of value and therefore should not be pursued as a model to characterize secondary RNA structure? No, not at all. It does suggest however that the model needs to contain more information in order to be discriminating. One way this can be achieved is by assigning weights to the vertices and edges based on the number of nucleotides, bases, and bonds in the respective stems and regions with unpaired bases. Karklin et al. [17] developed a labeled dual graph representation and defined a similarity measure using marginalized kernels. Using this measure they train support vector machine classifiers to identify known families of RNAs from random RNAs with similar statistics. They achieved better than seventy percent accuracy using these biologically relevant vertex and edge labels. Efforts to synthesize RNA molecules for various purposes such as novel drug applications as well as efforts to develop efficient genome-wide screens for RNA molecules from existing families may be aided by the graph representation in the RAG database when coupled with vertex and edge weighting schemes. Indeed, the authors have successfully used vertex weighted graphs to characterize the residue structure of amino acids in order to build a predictive model of binding affinity levels resulting from single point mutations [18]. Future work naturally points to using vertex weighted graphs for the characterization of a secondary RNA structure. Information revealed by the labeled dual graph representation which shows that a secondary RNA structure is not consistent with those known to be found in nature can be considered a valuable resource for biotechnological applications, automated discovery of uncharacterized RNA molecules, and computationally efficient algorithms that can be used in conjunction with other methods for RNA structure identification.
